# Insights from Pheromone Trap Catches in the Northern Part of the *Ips typographus* Range

**DOI:** 10.3390/insects17060610

**Published:** 2026-06-09

**Authors:** Andrey Selikhovkin, Nikita Mamaev, Maria Martirova, Nickolai Sedikhin

**Affiliations:** 1 Department of Forest Protection, Wood Science and Game Management, Saint Petersburg State Forest Technical University, Institutski Lane 5, 194021 St. Petersburg, Russia; a.selikhovkin@mail.ru (A.S.); masha2340350@yandex.ru (M.M.); 2 Zoological Institute of Russian Academy of Science, Universitetskaya Emb., 1, 199034 St. Petersburg, Russia

**Keywords:** European spruce bark beetle, pheromone monitoring, flight dynamics, climate factors

## Abstract

The European spruce bark beetle (*Ips typographus*) is the main pest of spruce in northwestern European Russia, particularly in the Leningrad Region. Its outbreaks occur quite frequently. However, the population dynamics of *Ips typographus* in this region remain poorly understood. The aim of this study is to identify the life cycle characteristics of the species based on data obtained using pheromone traps in the Leningrad Region, to clarify the influence of various factors, and to evaluate the effectiveness of this monitoring method. From 2022 to 2025, observations using barrier pheromone traps north and south of St. Petersburg at several points were carried out. There were 3 traps placed at each point. The traps were inspected at 5-day intervals from early May to late August. The dynamics of beetle flight was obtained based on standardized values of beetle catches. The relationship between beetle swarming and temperature was estimated based on calculated Growing Degree-Days for 2022, 2023 and 2025. A graphical representation of 5-day moving average for daily temperature, relative humidity, wind speed and atmospheric pressure in accordance with calculated swarming dynamics were illustrated. The spring mass flight of the parent generation correlated strongly with daily temperature, but no significant correlation was found with other factors or outside the spring period. Catches after spring flight did not reflect actual population levels. Pheromone traps reliably reflect population density only during the spring flight of the parent generation.

## Simple Summary

The European spruce bark beetle (*Ips typographus*) is the most significant pest of spruce in Europe. The population dynamics of this pest, and particularly the increase in its outbreaks and range expansion due to climate change, have been the subject of numerous studies. In the Leningrad Region (located in the northwestern part of European Russia, surrounding St. Petersburg), the main causes of spruce decline over the past 10 years have been recreational pressure, improper forest management, and extensive construction. These factors have contributed to an increase in the frequency of bark beetle outbreaks. The latest outbreak of this pest began in 2021. We tracked the population dynamics and development patterns of the bark beetle in this region. From 2022 to 2025, we monitored its population using barrier pheromone traps with lure Vertenol. The traps were located in blueberry spruce forests under the forest canopy. No tree felling was carried out in the observation sites. The spring swarming period depends significantly on weather conditions and occurs during the following periods: 2022–23.05–30.05; 2023–06.05–25.05; 2024-; 2025–16.05–25.05. The proportion of beetles captured after swarming was 22% or less, with one exception (51%). In 2025, after the spring swarming, only a few beetles were caught in traps. These values are consistent with those obtained by other authors in Scandinavia. The number of individuals captured in traps after the completion of spring swarming during univoltine development does not reflect the size of the young generation. The use of pheromone traps as a means of monitoring population density is effective only during the period of the first swarming of the parent generation. It was during this period that an increase in trapping intensity was noted with an increase in average daily temperatures. Subsequent trapping does not reflect the dynamics of population density. The data correspond to the results of other authors. We analyzed the relationship between flight dynamics, i.e., the number of beetles captured in traps, and climatic factors (temperature, wind, humidity, and pressure). A significant correlation was found only for average daily temperature values during the swarming of the parental generation. No relationship with the number of beetles captured was observed for other climatic factors. No effect of climatic factors on swarming was detected either for individual periods (except for the swarming of the first generation) or when analyzing the entire dataset over several months of observations.

## 1. Introduction

The European spruce bark beetle (*Ips typographus*) is the most significant phloem-feeding pest in Europe. The population dynamics of this pest, and especially its increased outbreak activity and range expansion due to climate change, have been the subject of numerous studies [1,2,3,4,5,6]. One method for monitoring population dynamics and population control of the European spruce bark beetle involves pheromone traps, with (4S)-cis-verbenol as the key component [7]. The use of this method has little or a highly debatable effect on reducing the population of the European spruce bark beetle [4,8], and in some cases it may increase the frequency of attacks [9]. Even with a high density of traps, only 3 to 30% of beetles are caught [10]. The deployment of 270,000 traps in Sweden did not have a significant impact on the *I. typographus* outbreak [11]. Conversely, the use of pheromone traps for monitoring and predicting the population dynamics of the European spruce bark beetle is the most common and highly effective method [12,13,14]. It is well known that the number of the first and sister generations is defined by the sum of effective temperatures, or Growing Degree Day (GDD) [1,15,16,17,18]. However, interpreting the data requires considering that pheromone trap catches depend on numerous factors—temperature, exposure, light conditions, availability of suitable host trees near the traps, wind direction and strength, among others [4,9,12,14,19,20]. Consequently, the dynamics of bark beetle catches in pheromone traps may not reflect the emergence of a second generation or the number of sister generations [6,9,14,21,22]. However, in Russian forest protection practice, pheromone traps have been adopted and are recommended for reducing pest numbers and, in particular, for monitoring their populations [19,23,24]. In particular, the number of beetles caught during the mass flight period is used as a criterion for the probability of an outbreak focus occurring or already being present, and for making decisions on implementing forest protection measures. For example, if a single trap captures 200–1000 beetles per day or 3000–8000 per month, this signals an outbreak threat and necessitates measures to reduce the European spruce bark beetle population [23]. Recent studies confirm that population density estimates based on trap catches are representative, particularly when conducted during the mass flight period in May and June. Moreover, the number of beetles caught in traps during the second half of the growing season has a weak dependence on population density and is determined by various specific factors [6,14,22].

Outbreaks of *Ips typographus* in the forests of the Leningrad Region and St. Petersburg occur quite frequently [15]. In 2021, another outbreak began, during which we registered bivoltine development of the beetle for the first time in our region [25]. In 2022, we conducted monitoring of flight dynamics using pheromone traps [15], which was continued in 2023, 2024, and 2025. Similar monitoring studies had previously been conducted mainly in more southern regions of Russia [19,20,24,26,27]. Several studies were carried out at local sites in Karelia [28,29,30]. This allowed for some generalizations to be made for the entire Republic of Karelia [30]. In the Republic of Karelia, as in the Leningrad region, the Forest Protection Center (a branch of the Russian Forest Protection Agency) conducts periodic monitoring of the *Ips typographus* population using pheromone traps. The quantitative results of these observations, however, are inaccessible. Studies of the effectiveness of pheromones were conducted at the Tsarskoye Selo State Museum-Reserve [31]. Regular monitoring of *Ips typographus* population dynamics using pheromone traps over several growing seasons has not previously been conducted in the Leningrad Region. Consequently, there are no actual data on the flight and development dynamics of the bark beetle in this region. This prevents the consideration of regional characteristics of the development dynamics of this pest in the Leningrad Region and St. Petersburg when conducting research on the biology of the bark beetle and planning forest protection measures.

The purpose of this study is to assess the extent to which the results of *Ips typographus* beetle captures using pheromone traps reflect actual biological processes (swarming, population density, and weather-related factors) in the Leningrad Region. The aims included (1) determining the life cycle characteristics of the bark beetle species based on data obtained using pheromone traps and (2) analyzing the relationship between flight parameters and climatic factors (temperature, wind speed, humidity, and atmospheric pressure).

## 2. Objects and Methods for Obtaining Data

**Figure 1:**
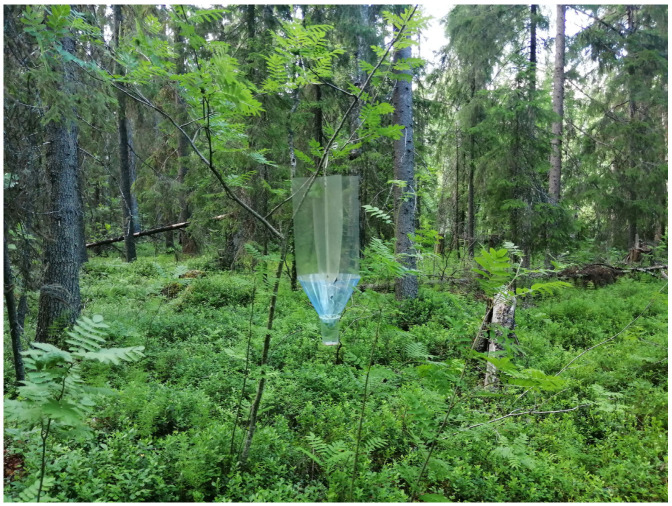
Pheromone traps (produced by All-Russian Plant Quarantine Center (VNIIKR)) (photo A. Selikhovkin, 2024, 59.43356, 30.69228).

**Figure 2:**
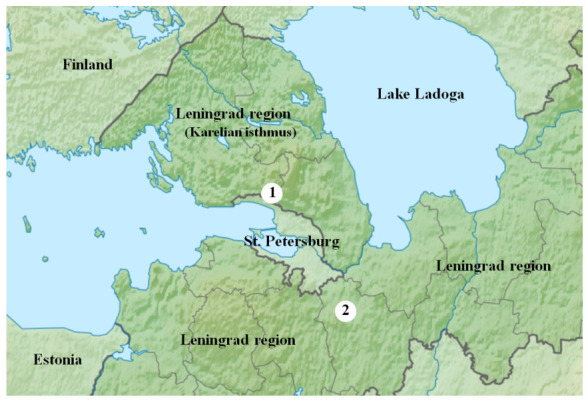
Locations of pheromone traps for *Ips typographus* in the Leningrad region: Location 1: 60.23446, 29.62478 in 2022 and 2023; 60.22215, 29.79213 and 60.17874, 29.78806 in 2024; Location 2: 59.43356, 30.69228 and 59.42744, 30.69959 in 2023 and 2025; 59.43356, 30.69228 and 59.41733, 30.69873 in 2024.

Bark beetle captured using barrier pheromone traps produced by All-Russian Plant Quarantine Center (VNIIKR); Lure “Vertenol” (Bykovo, Moscow Region, Russia) (Figure 1). *I. typographus* beetles were captured annually from 2022 to 2025. Traps were placed on the territory of the Leningrad region in approximately the same locations: north of St. Petersburg on the Karelian Isthmus, near the settlement of Roschino (Location 1), and to the south in Tosnensky district of the Leningrad region, near settlement Lisino-Korpus (Location 2) (Figure 2). There were two observation points at Location 2 in all years, and one or two points at Location 1 (Figure 2). At least three traps were always set in each point. The traps were located in blueberry spruce forests under the forest canopy, 1.3 m above the ground. Spruce accounted for 80–90% of the forest stand. The average age of the stand was 80–90 years. Tree density varied from 310 to 360 trees per hectare. No tree felling was carried out in the observation sites. The proportion of dead spruce trees that died as a result of the reproduction of the bark beetle did not exceed 10% in all years of observation.

Traps were inspected at 5-day intervals from early May to late August. In 2024, traps on the Karelian Isthmus were placed at two points located slightly south of the sites used in other years (Figure 2) and beetles were collected from traps at 14-day intervals [32].

Data on climatic factors were obtained from a publicly available online resource [33] for the five-month period from April to August inclusive for each year of observation. We analyzed the dependence of flight on temperature, wind, humidity, and atmospheric pressure. We calculated the daily average temperatures, humidity, and pressure from data recorded at three-hour intervals throughout the day. If the source weather archive contained wind speed values exceeding the measured average, the daily average wind speed was recalculated as the mean of all recorded wind speed values.

Publicly available weather data were obtained from the meteorological stations closest to the trap locations: for the Location 1—from the Lisiy Nos station (60.0115, 29.9699) in 2022 and from the Roshchino station (60.2416, 29.6219) in 2023; for the Location 2—from Belogorka (59.3484, 30.1331). After processing the data from the meteorological stations, we found only minimal differences in the values of the main climatic parameters studied. Therefore, in this article and in the analysis of the relationship between beetle swarming and climatic factors, we used data from only one station—Belogorka, which is closest to our study site in Location 2.

## 3. Data Processing Methods

The Growing Degree-Days (GDD) were calculated for each observation year. The formula for calculating GDD for particular day was as follows:

$$GDD\;=\;T_{mean}\;-\;T_{base}\;,$$

where $T_{mean}\;=\;\frac{\left(T\;in\;00:00AM\;+\;T\;in\;3:00AM\;+\;\ldots \;+T\;in\;6:00PM\;+\;T\;in\;9:00PM\right)}{8}$—daily average temperature measured on 8 values at a specific hour, °C; $T_{base}\;$—threshold values of temperature required for development of the first generation, °C. The threshold air temperatures for the development of different life stages of *Ips typographus* vary from 5 °C (flight of first-generation beetles) to 10.6 °C (egg development). Accordingly, the GDD required for the development of the first generation was calculated for two threshold values that are commonly used in studies from similar climatic zones: 5.0 °C and 8.3 °C [16,22,34,35]. For result comparison we used GDD data from central Sweden: the GDD was 666 degree-days for the 5.0 °C threshold, and it was 420 degree-days for 8.3 °C [22].

The swarming period was defined as the time interval during which the average daily number of beetles caught per trap exceeded ten. These intervals were clearly distinguishable in all catch sites. Relative values of beetles caught were used for correlation and regression analyses instead of absolute values. The number of beetles from all traps at each site was averaged and standardized for each check period. The values of standardized scale ($z_{i}$) were determined as:

$$z_{i}=\frac{x_{i}-\bar{X}}{\sigma_{i}}\;,$$

where $x_{i}$—the number of beetles of , $\bar{X}$—mean value, $\sigma_{i}$—standard deviation, estimated from the dataset of beetles caught in 2022, 2023, and 2025 at the observation points. Swarming peaks were determined based on the dynamics of standardized catch values. A moving average of the standardized catch values was calculated for each day from May to August using values from a 5-day interval (current day ± 2 days). A moving average of mean daily temperatures was calculated the same way. This transformation was performed to achieve a more informative visual representation of the data.

Pearson (cor), Spearman (rho), and Kendall (tau) correlation coefficients were calculated to assess the relationships between beetle flight activity and climatic factors, and multiple linear regression analysis was also performed [36,37]. Calculations of correlation coefficients and paired regression analyses were carried out using the Data Analysis ToolPak in Microsoft Excel 2016 and the R statistical environment (Version 4.3.3) [38]. Correlation was assessed using data on the number of beetles caught for each full observation period, as well as for the initial phases of the first swarming peak up to the point when catches began to decline.

## 4. Results

**Figure 3:**
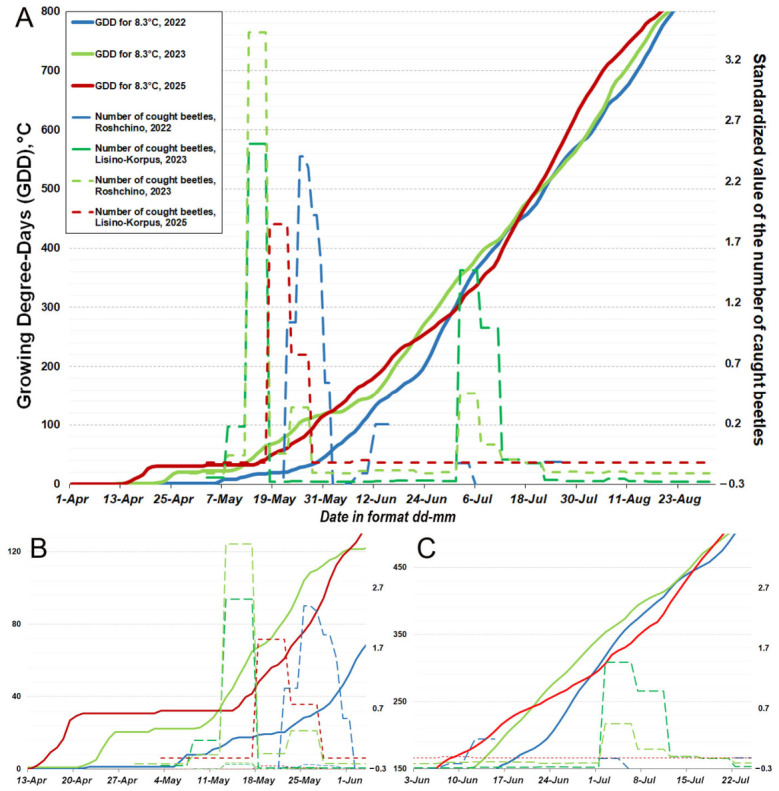
Dynamics of beetle swarming based on standardized values and the GDD for 2022, 2023, and 2025: (**A**) for the period from 1 April to 28 August; (**B**) from 13 April to 3 June; (**C**) from 3 June to 25 July. The designations in parts (**B**,**C**) are as shown in part (**A**). GDD data are from the Belogorka weather station.

**Figure 4:**
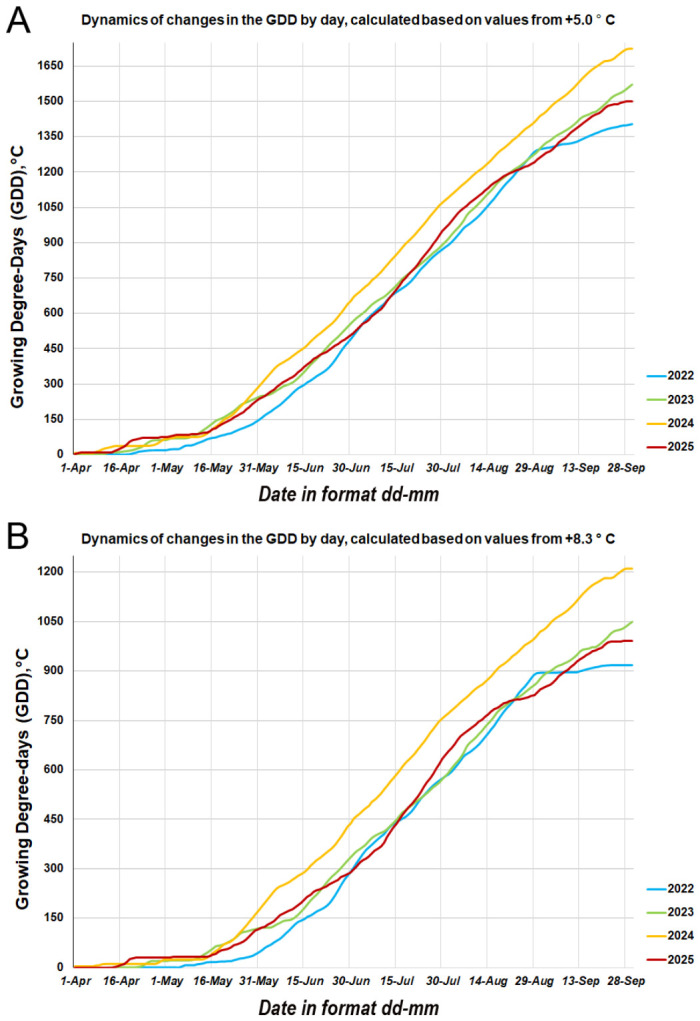
Dynamics of the GDD changes by day for two threshold values based on data from the Belogorka weather station: (**A**) calculations are presented for the 5.0 °C average daily temperature threshold; (**B**) calculations are presented for the 8.3 °C average daily temperature threshold.

The GDD graph and the plot of standardized catch values (Figure 3) clearly indicate a temporal relationship between temperature and the onset of flight activity. Heat supply during the April–September period was the lowest in 2022 and the highest in 2024 (Figure 4). In simplified terms, the later heat accumulation occurs, the later the swarming flight of the parent generation begins. The GDD required for the start of the swarming of the parent generation was as follows:

- In 2022 (20–23 May): for 5.0 °C—84–97, for 8.3 °C—19–20;
- In 2023 (10–15 May): for 5.0 °C—81–124, for 8.3 °C—22–25;
- In 2025 (18–20 May): for 5.0 °C—122–137, for 8.3 °C—39–52.

The number of beetles caught was highest during the spring swarming of the parent generation in all cases. This period varied substantially in both start date and duration. In 2023, this period started earliest, on 6 May, in both locations; in 2025, it began on 16 May; and in 2022, it started latest, on 23 May, but was the most numerous in terms of beetle catches. The end of the spring swarming in 2024 occurred on 27 May, with daily catches from 24 to 27 May averaging 12 beetles per trap (Table 1). In all observed periods, swarming began after the daily average temperature reached 10 °C. This shift in the timing of swarming onset corresponds to the dynamics of spring temperatures (Figure 3B). Data on beetles capture in 2024 were not included in the figures because the longer trap check interval resulted in incomplete data.

In 2022 and 2023, in addition to the spring swarming, we observed several other periods when beetles were caught in large numbers in the traps. According to our data, the second generation swarming on the Karelian Isthmus occurred from 27 June to 7 July in 2022 and from 29 June to 19 July in 2023. In 2023, the second generation swarming in the Tosnensky District occurred at exactly the same time as on the Karelian Isthmus, similarly to the spring swarming of the first generation (Figure 3C). In 2023, the GDD for the period from 29 June to 4 July (the second generation flight period) ranged from 324 to 366 degree-days for the 8.3 °C threshold and from 542 to 600 degree-days for the 5.0 °C threshold. In 2023 and 2024, field surveys of infested trees were conducted, which confirmed the timing of the second generation swarming [2,15,25,32].

In 2024 and 2025, no other periods of active catches were recorded after the completion of the parent generation flight. After 25 May 2025 (the end of the spring swarming), only two beetles were caught during the entire remaining observation period. However, infestation of wind-felled trees was observed in the area of the trap site at coordinates N59.42744°, E30.69959° (Location 2). This allowed us to determine the timing of re-colonization by the parent generation (12–13 July 2025) and the onset of second generation swarming flight (3 August 2025). Before the onset of frost, this development ended at the larval or pupal stage. All individuals died during the winter. This is quite natural, since this species successfully diapauses only at the imago stage [1,39]. In another group of traps, located in a denser spruce stand, wind-felled trees were not colonized.

It should be noted that the number of beetles captured after the end of the spring swarming is, in most cases, much lower than that of the parent generation. Thus, the proportion of beetles caught after the swarming is often less than 50% (Table 2). Only in 2023, at Location 2, was the number of beetles caught during the spring swarming identical to the number caught afterwards. However, this indicator varies significantly for each individual trap.

Analyses of data from 2022 and 2023 on the relationships between swarming dynamics and temperature, wind speed, humidity, and atmospheric pressure showed that a positive correlation was observed only between the standardized number of captured beetles and mean daily temperature. Moreover, this relationship was significant for the period from the zero values preceding the start of swarming to the peak values (n = 9, cor = 0.76, rho = 0.8, tau = 0.61, *p*-value < 0.05). The correlation coefficients increased when similar calculations were performed using moving average values of mean daily temperature (n = 9, cor = 0.79, rho = 0.85, tau = 0.72, *p*-value < 0.05). Thus, the relationship between the number of beetles caught and temperature dynamics was established only for the parent generation swarming period.

**Figure 5:**
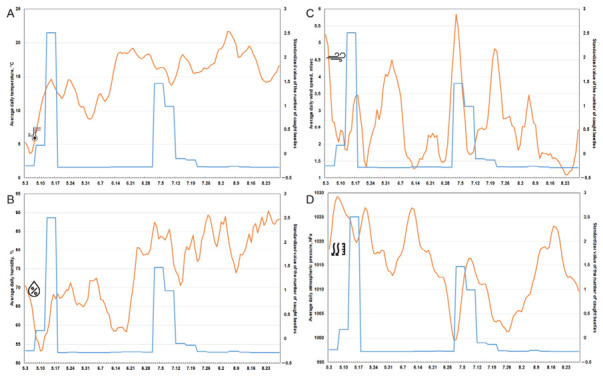
Graphical representation of swarming dynamics of beetles trapped near Lisino-Korpus (Uchebno opytnoye lesnichestvo, Tosnensky District) based on standardized values and the 5 day moving average of climatic factors: (**A**) mean daily temperature (°C); (**B**) mean daily relative humidity (%); (**C**) mean daily wind speed (m/s); (**D**) mean daily atmospheric pressure (hPa). The X-axis labels represent the date in the format mm-dd. The presented data are from the Belogorka weather station for the 2023 observation period.

A significant correlation between the number of catches and other climatic factors was not found. Also, no significant correlation was found with any of the other climatic factors when analyzing the 4-month dataset (*p*-value > 0.05) (Figure 5). Similar results were obtained from regression analysis. Linear models could not reliably describe the dependence of beetle flight on climatic factors (*p*-value > 0.05). None of the factors had a significant effect on beetle flight, with R^2^ values close to zero. In the May swarming data subset, only temperature was a significant factor (Pr > |t| = 0.03), but not at the predetermined significance level, as the *p*-value was 0.104. The R^2^ value was approximately 0.8. The GDD at the onset of the May swarming ranged from 60 to 80.

## 5. Discussion

European spruce bark beetle outbreaks in northern forests are most often associated with wind-felled or drought-stressed trees [39,40,41]. In the Leningrad region wind-falls over large areas have not been observed in the last 10 years. However, in the spruce forests of this region, small outbreaks of the bark beetle comprising approximately 5–15 infested trees are constantly present. Their occurrence is associated with local weakening of tree stands due to improper forest management, high recreational loads, and road construction that disrupts the hydrological regime. In 2021, extremely warm June and July provided the opportunity for the formation of a second generation and three sister generations. The population density increased by two orders of magnitude in one season. Having started in 2021, this outbreak of European spruce bark beetle in the Leningrad region ended in 2023 (Table 1) [15,32].

In all observed periods, swarming began after the daily average temperature reached 10 °C. However, daily temperature exhibits natural fluctuations between day and night, and the spring and early summer periods in Northwestern Russia tend to have unstable weather conditions. This undoubtedly influences the termination of diapause. Shifts in the swarming flight periods of the parent and new generations depending on temperature dynamics are an expected result [4,6,22,34].

It should be noted that the daily average temperature of 10 °C is meaningless in itself. It is shown graphically that a certain amount of heat accumulation is necessary for the start of the parent generation swarming period, and this accumulation, depending on its rate and timing, determines the onset of flight (Figure 3B). We found that different GDD values correspond to the dates of swarming onset in different observation years: 20–80 degree-days for 2022–2023 and 40–120 degree-days for 2025. However, the dates of flight onset differ independently of GDD value. One might suggest that a rise in mean daily temperature to 8–10 °C serves as a trigger point. However, in this context, the “awakening” of beetles is dependent on a prolonged combination of temperature regimes, including periods with values lower than 8–10 °C. It is not yet advisable to speak of absolute temperature thresholds after which swarming should begin, especially with mention of GDD. If diapause termination is related to temperature dynamics, it is a fractal dynamic process, which makes it difficult to predict. It remains to be determined how many consecutive days the minimum required temperature must persist, and how this period changes with temperature fluctuations. In 2025, the GDD was high; however, a prolonged cold spell following the April warming may have delayed the onset of parent generation swarming compared to 2023. In 2022, GDD accumulation was more even, but the rate of temperature increase was minimal, which is why, according to observations, flight onset occurred the latest. In the study region, as in Sweden and Norway, air temperatures can drop to critical levels in May, significantly decreasing beetle activity. The timing of the parent generation swarming in northern populations may depend on temperature conditions [39,42].

The observed significant simultaneous increase in both catch numbers and daily average temperature in May at the observation points can be considered an indication of the onset of parent generation swarming. However, this correlation was found only for a short period with a small set of measurements, i.e., from zero to peak values during the swarming of the parent generation (n = 9, cor = 0.76, rho = 0.8, tau = 0.61, *p*-value < 0.05). This period was subjectively separated from all others. In subsequent intervals, a decrease in temperature, unlike flight activity, is less likely. Accordingly, for other time intervals, as well as for the entire observation period as a whole, no significant relationships with the studied climatic factors were found.

The studies by N.I. Lyamtsev and V.N. Kolobov [20,26] also present data on the dynamics of catches and average daily temperatures. During the spring swarming of the parent generation, the graphs show a dependence of the number of trapped *I. typographus* beetles on temperature fluctuations. However, contrary to the authors’ claims, this dependence is not observed during subsequent catches. Unfortunately, the authors did not perform a statistical analysis of their data. Based on our results, it should be assumed that the seasonal dynamics of weather factors do not significantly affect pheromone trap catches.

Regarding the summer swarming of the first generation, according to our data, it was observed in 2023 in Lisino and Roshchino, and partially in 2022 in Roshchino. In 2024 and 2025, a very small number of beetles were recorded after the spring swarming, approaching minimum values on the standardized scale. For the dates when an increase in beetle catches was recorded in early July 2023, the GDD corresponded to approximately 300–360 (for the 8.3 °C development threshold) and 500–600 (for the 5 °C threshold) degree-days. Therefore, it can generally be stated that the GDD required for the development of the first generation corresponds to the range of 666 (5 °C threshold) to 418 (8.3 °C threshold) degree-days established earlier for northern and central Sweden [22], and 557 in Tara Mountain in western Serbia [41]. These values vary significantly depending on illumination. For southern Sweden, during different observation periods, sun-exposed and shaded conditions ranged from 620–693 and 526–677 DD (5 °C), respectively [40]. We did not take this factor into account. All our traps were located under the forest canopy.

The dynamics of GDD accumulation do not change depending on the temperature chosen for the calculation (Figure 4). For GDD calculated with the 5 °C threshold, the absolute values simply increase. Moreover, the swarming of the first generation recorded in this study occurred during the same periods in 2022 and 2023, regardless of the GDD values. Based on the foregoing, a reasonable question arises about the overall expediency of calculating this parameter. This is corresponded to a study of the population dynamics of European spruce bark beetles in Sweden. The use of the GDD parameterization of the flight dynamics model is of secondary importance, as the average daily temperature in the spring after swarming is above the highest proposed temperature threshold [40].

In northern regions, such as Scandinavia, Karelia, and the Leningrad region, the bark beetle typically has a single generation. Larvae and pupae that remain under the bark overwinter only survive in mild winters [43]. Thresholds of winter temperatures are −13 °C and −17 °C for larvae and pupae [44]. At subzero winter temperatures in Scandinavia and northern and central Russia, only beetles successfully overwinter [1,44,45]. In southern Sweden, a portion of the population may form a second generation in warm years [42]. This phenomenon was first recorded in the Leningrad Region, including its northern part on the Karelian Isthmus [15,25]. Previously, bivoltine development was observed for *I. typographus* and *Ips duplicatus* (Sahlberg, 1836) in Estonia, where climatic and weather conditions are similar to those in the Leningrad Region [46]. During univoltine development of the bark beetle (the predominant developmental mode in northern forests), the effect of pheromone on the young generation of emerging beetles is significantly reduced. In this case, young beetles are not motivated by the scent of lure, as they do not initiate mating. They undergo additional feeding under the bark of infested or severely weakened trees and then select a site for overwintering. Only a portion of the sister generation falls into pheromone traps.

The proportion of beetles caught in traps after the spring swarming relative to the total number of beetles caught in northern regions is relatively small. In more southern regions, this proportion can be significantly higher, ranging from 50 to 80% [13,20,26]. Given the bivoltinism of these populations and the presence of several sister generations, this is quite explainable. In our case, this ratio varied from 0.6 to 51.2%. This was also demonstrated by long-term observations conducted in Sweden. The ratio of young beetles to the total number of beetles captured was also low, ranging from 0.2 to 18% [22]. In 2025, due to the low GDD, the development of the young generation was delayed. Almost no beetles were caught in traps after the swarming, but the emergence of the young generation was successful in the colonized trees in the capture area. Thus, the number of individuals captured in traps after the completion of spring swarming during univoltine development does not reflect the size of the young generation.

## 6. Conclusions

During the first period of swarming of the parent generation of *Ips typographus*, the intensity of capture increases with the growth of the average daily temperature. A significant correlation was found only between the number of captured beetles and average daily temperature values during the swarming period of the first parental generation. There was no correlation between this indicator and the dynamics of other climatic factors (wind, humidity, and pressure). No influence of climatic factors on swarming was detected either for individual periods (except for the swarming of the first generation) or when analyzing the entire dataset over several months of observations.

Using pheromone traps to monitor the density of a univoltine *Ips typographus* population is effective only during the first swarming period of the parental generation. Subsequent capture of beetles using pheromone traps does not reflect population density dynamics.

For further study of the cyclical processes of flight dynamics and the impacts of climatic and other environmental factors on them, complex hypotheses about the causes and factors of the development of different generations must be tested based on large datasets.

## Figures and Tables

**Table 1:** Periods of swarming and average number of beetles caught per trap per day (for captured ≥10 specimens per trap per day).

Year	Location 1		Location 2	
	Period	Specimens per Trap per Day	Period	Specimens per Trap per Day
2022	23.05–30.05	585	-	-
	03.06–13.06	68	-	-
	27.06–07.07	20	-	-
	17.07–01.08	24	-	-
2023	06.05–25.05	654	06.05–15.05	155
	05.06–19.06	14	16.05–29.06	<10
	30.06–19.07	95	30.06–19.07	137
2025	-	-	16.05–25.05	24
	-	-	03.08 *	0

Note: *—start of new generation emergence according to observations on wind-felled trees; “-”—no data.

**Table 2:** Ratio of the number of specimens caught after the end of the spring swarming to the total number of specimens caught in pheromone traps.

Year	Location 1			Location 2		
	Total per Season, Specimens	After Swarming, Specimens *	Ratio, %	Total per Season, Specimens	After Swarming, Specimens *	Ratio, %
2022	40,585	8860	21.8%	-	-	-
2023	20,085	4355	21.6%	6546	3356	51.2%
2024	-	-	-	399	53	13.2%
2025	-	-	-	291	2	0.6%

Note: *—the number of beetles “after swarming” was counted from the first observation of zero catches in traps after the spring swarming of the parent generation. This occurred universally in the first ten days of June; “-”—no data.

## Data Availability

The original contributions presented in this study are included in the article. Further inquiries can be directed to the corresponding authors.

## Abbreviation

The following abbreviation is used in this manuscript:

GDD	Growing Degree Day
